# Aging STINGs: mitophagy at the crossroads of neuroinflammation

**DOI:** 10.1080/15548627.2024.2322421

**Published:** 2024-02-27

**Authors:** Juan Ignacio Jiménez-Loygorri, Patricia Boya

**Affiliations:** a Department of Cellular and Molecular Biology, Centro de Investigaciones Biológicas Margarita Salas, CSIC, Madrid, Spain; b Department of Neuroscience and Movement Science, Faculty of Science and Medicine, University of Fribourg, Fribourg, Switzerland

**Keywords:** Inflammation, mitochondria, mtDNA, parkin, PINK1, retina

## Abstract

Loss of proteostasis and dysregulated mitochondrial function are part of the traditional hallmarks of aging, and in their last revision impaired macroautophagy and chronic inflammation are also included. Mitophagy is at the intersection of all these processes but whether it undergoes age-associated perturbations was not known. In our recent work, we performed a systematic and systemic analysis of mitolysosome levels in mice and found that, despite the already-known decrease in nonselective macroautophagy, mitophagy remains stable or increases upon aging in all tissues analyzed and is mediated by the PINK1-PRKN-dependent pathway. Further analyses revealed a concomitant increase in mtDNA leakage into the cytosol and activation of the CGAS-STING1 inflammation axis. Notably, both phenomena are also observed in primary fibroblasts from aged human donors. We hypothesized that mitophagy might be selectively upregulated during aging to improve mitochondrial fitness and reduce mtDNA-induced inflammation. Treatment with the mitophagy inducer urolithin A alleviates age-associated neurological decline, including improved synaptic connectivity, cognitive memory and visual function. Supporting our initial hypothesis, urolithin A reduces the levels of cytosolic mtDNA, CGAS-STING1 activation and neuroinflammation. Finally, using an *in vitro* model of mitochondrial membrane permeabilization we validated that PINK1-PRKN-mediated mitophagy is essential to resolve cytosolic mtDNA-triggered inflammation. These findings open up an integrative approach to tackle aging and increase healthspan via mitophagy induction.

Defective autophagy has historically been linked to the aging process, and it becomes particularly relevant in the context of post-mitotic tissues where intracellular debris and undigested lysosomal products accumulate over a lifetime. Aberrant autophagic degradation has also been linked with increased incidence of age-associated pathology such as Alzheimer disease, cancer or age-related macular degeneration (AMD). Our previous work indeed has shown that macroautophagic flux decreases with age in the retina, but alternative and more selective pathways such as chaperone-mediated autophagy (CMA) can be upregulated to sustain basal proteostasis levels. Because mitochondrial dysfunction was also considered one of the main hallmarks of aging, we set out to study whether its selective upcycling via mitophagy was also stalled upon aging.

There is an extensive body of work regarding mitophagy regulation during physiological aging, but the lack of proper tools and complex data interpretation led to contradictory results even from parallel studies analyzing the same cell type or tissue. Taking advantage of the tandem fluorescence *mito*-QC reporter mouse (mCherry-GFP-FIS1[101–152] in a C57BL/6J background), we previously described very high levels of mitophagy in the adult retina to the extent where it accounts for almost all ongoing macroautophagic degradation within the photoreceptor-containing outer nuclear layer. In our recent work [1], we compared mitolysosome content in young (6–8 months) and old (22–26 months) mice and, surprisingly, found that mitophagy levels are increased in the old cohort. More importantly, this observation was not restricted to the retina as mitophagy is also upregulated in the aged kidney, brain, RPE, cerebellum and liver; we also analyzed pancreas, spleen, muscle, heart and lung and found that, even though mitophagy levels do not increase, they remain stable throughout aging. Increased phosphorylation of ubiquitin at its Ser65 residue revealed that it is mediated by the PINK1-PRKN-dependent mitophagy pathway. We observe no changes in the levels of receptor-mediated mitophagy effectors or cardiolipin translocation to the outer mitochondrial membrane, involved in lipid-mediated mitophagy. We initially hypothesized that selective mitophagy upregulation could be due to defective mitochondrial function but no changes are observed regarding mitochondrial mass or oxidative phosphorylation proteins. However, electron microscopy reveals signs of mitochondrial herniation and membrane rupture.

We performed an untargeted transcriptomic analysis of the aged retina to further dissect this phenomenon and were surprised to find that the top upregulated pathways are involved in inflammatory type I interferon response; these results are puzzling as the neuroretina is shielded by the blood-retina barrier (BRB). Anti-DNA immunofluorescence analysis helped us identify cytosolic DNA foci in the retina that localize next to mitochondria-rich regions, and subcellular fractionation coupled with qPCR revealed that these foci correspond to mtDNA. Immunoblotting shows a marked increase in CGAS-STING1 levels and downstream IRF3 signaling activation, that explain the observed upregulation of inflammatory pathways. All these findings are also replicated in other mouse organs as well as in primary dermal fibroblasts from young and old human donors, indicating that this phenomenon is conserved cross-organ and cross-species.

We pivoted from our initial hypothesis and proposed that mitophagy upregulation during aging could then be a coping mechanism to deal with sterile inflammation caused by cytosolic mtDNA. The natural compound urolithin A (UA) has been described as a potent PINK1-PRKN-dependent mitophagy inducer in animal models and humans, with a favorable pharmacokinetic profile in preliminary clinical trials. Mass spectrometry analysis in plasma and perfused brains validated for the first time that UA is able to cross the blood-brain barrier, and therefore the slightly-more permeable BRB, and enter the central nervous system. We performed an intervention study in young and old mice, whereby they received 2.3 mg/kg/day UA during eight weeks or the corresponding dose of vehicle. UA is able to induce mitophagy in both cohorts, and also increases mitochondrial biogenesis in the old group. Old mice that received UA show no signs of cytotoxicity and present greater cognitive memory, visual function and synaptic connectivity. Most importantly, UA reduces mtDNA leakage in the retina of old mice, activation of the CGAS-STING1-IRF3 transcriptional program and age-associated neuroinflammation, characterized in the retina by reactive astrogliosis and microglial infiltration.

Because we observed that UA is simultaneously stimulating mitochondrial biogenesis in old mice, we used an *in vitro* model of mtDNA leakage in tissue-relevant human ARPE-19 cells to dissect whether biogenesis is also required to resolve CGAS-STING1 activation. Treatment with the BCL2 inhibitor ABT-737, in combination with the caspase inhibitor Q-VD-OPh, triggers the formation of BAX and BAK1 pores in the mitochondrial membrane, allowing the release of mtDNA to the cytosol with no apoptotic cell death. Replicating our findings in mice and human fibroblasts, free cytosolic mtDNA induces PINK1-PRKN-dependent mitophagy and, surprisingly, it can be abrogated by co-treatment with the CGAS inhibitor G140. These results point to a reciprocal regulation between the CGAS-STING1 axis and mitophagy activation that warrants further exploration. Finally, to ascertain whether biogenesis was also contributing to mtDNA clearance, we inhibited mitochondrial protein synthesis (chloramphenicol) and/or PINK1-PRKN-dependent mitophagy (*PINK1;PRKN*-siRNA) in this system. Mitophagy inhibition leads to an accumulation of cytosolic mtDNA in UA-treated cells and we concluded that biogenesis is dispensable for the beneficial effects of UA in our experimental setup.

In conclusion, we have demonstrated that PINK1-PRKN-dependent mitophagy increases with age, in spite of a decrease in nonselective macroautophagy, as a counteracting mechanism to curtail mtDNA leakage into the cytosol and limit neuroinflammation (Figure 1). This process is amenable for pharmacological modulation using the mitophagy inducer UA, which is able to reduce age-associated neuroinflammation and increase healthspan. Thus, mitophagy induction represents a novel druggable target to reduce sterile inflammation triggered by cytosolic mtDNA during physiological aging.

**Figure 1.**
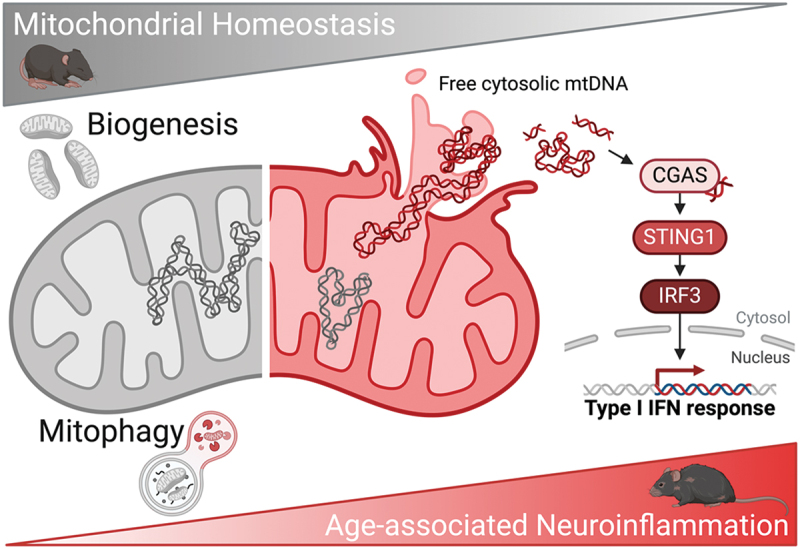
Mitophagy is a protective mechanism against age-associated mitochondrial dysfunction, mtDNA leakage and subsequent CGAS-STING1-mediated neuroinflammation. Diagram created with BioRender.com.
